# Quality-by-Design Approach to Process Intensification of Bioinspired Silica Synthesis

**DOI:** 10.1021/acssuschemeng.3c07624

**Published:** 2024-03-08

**Authors:** Joseph R. H. Manning, Carlos Brambila, Kabir Rishi, Gregory Beaucage, Gemma-Louise Davies, Siddharth V. Patwardhan

**Affiliations:** † Green Nanomaterials Research Group, Department of Chemical and Biological Engineering, University of Sheffield, Sheffield S1 3JD, United Kingdom; ‡ Department of Chemistry, University College London, London WC1H 0AJ, United Kingdom; § Department of Chemical and Materials Engineering, University of Cincinnati, Cincinnati, Ohio 45221, United States

**Keywords:** manufacturing, scale-up, green chemistry, sustainability

## Abstract

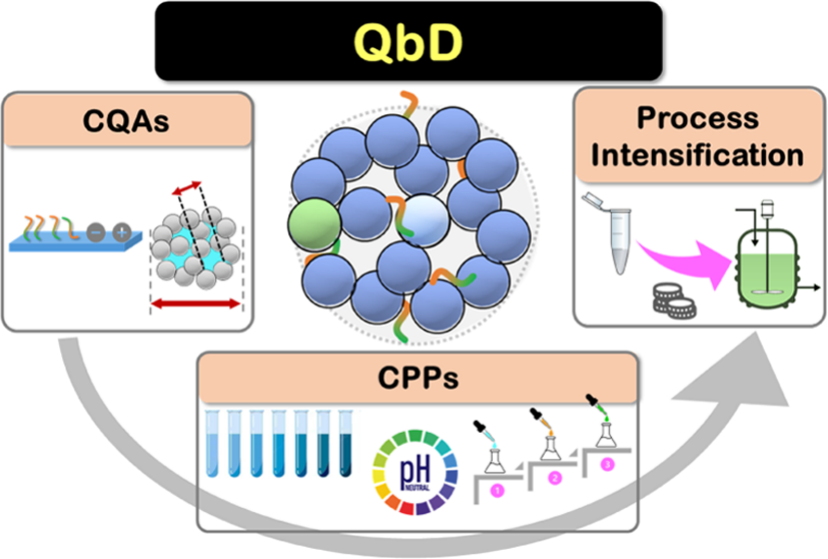

Characterizing nanomaterials is challenging due to their macromolecular nature, requiring suites of physicochemical analysis to fully resolve their structure. As such, their synthesis and scale-up are notoriously complex, especially when compared to small molecules or bulk crystalline materials, which can be provided a unique fingerprint from nuclear magnetic resonance (NMR) or X-ray diffraction (XRD) alone. In this study, we address this challenge by adopting a three-step quality-by-design (QbD) approach to the scale-up of bioinspired silica nanomaterials, demonstrating its utility toward synthesis scale-up and intensification for this class of materials in general. First, we identified material-specific surface area, pore-size distribution, and reaction yield as critical quality attributes (CQAs) that could be precisely measured and controlled by changing reaction conditions. We then identified the critical process parameters (CPPs) controlling bioinspired synthesis properties, exploring different process routes, incorporating commercial reagents, and optimizing reagent ratios, comparing silica properties against original CQA values to identify acceptable limits to each CPP. Finally, we intensified the synthesis by increasing reagent concentration while simultaneously incorporating the optimized CPPs, thereby modifying the bioinspired silica synthesis to make it compatible with existing manufacturing methods. We increased the specific yield from ca. 1.1 to 38 g/L and reduced the additive intensity from ca. 1 to 0.04 g/g product, greatly reducing both synthesis cost and waste production. These results identify a need for mapping the effects of critical process parameters on material formation pathways and CQAs to enable accelerated scale-up and transition from the lab to the market.

## Abstract 2

A schematic of the quality-by-design workflow: definition of critical quality attributes leads to identification of critical process parameters, facilitating process intensification

## Introduction

Nanomaterial synthesis and scale-up are notoriously complex, chiefly because of stringent process requirements needed to achieve their key properties.^1^ These requirements lead to very high environmental costs, with E-factors (mass of waste produced per mass of product)^2^ typically orders of magnitude higher for nanomaterials compared to fine or pharmaceutical chemicals.^3^ Nanomaterials are also more complex to characterize compared to small molecules or bulk crystalline materials, both of which can be dispositively identified using nuclear magnetic resonance or single-crystal X-ray spectroscopy, necessitating a broad array of chemical microanalyses to fully identify a specific formulation.^4^ Indeed, the OECD Working Party on Manufactured Nanomaterials^5^ has recognized that to fully describe nanomaterials, a range of physicochemical properties are required, e.g., chemical composition, surface chemistry, zeta potential, aggregation, particle sizes, crystallinity, and porosity. Although each of these techniques can be applied to nanomaterial characterization, few have standardized protocols for routine nanoparticle analysis.

Although seemingly unrelated, these two aspects of nanomaterials—wasteful synthesis methods and complex structure—combine to form significant barriers to process scale-up and intensification.^6^ Complete characterization of the nanomaterial structure and performance is typically performed only for samples synthesized using laboratory synthesis methods, meaning that samples synthesized with manufacturing-compatible methods cannot be guaranteed to exhibit the same behavior without dedicated analytical studies. These barriers therefore represent a significant risk to the translation of nanomaterial technologies from the laboratory to the market, compounding the already large “valley of death” to commercial adoption.^7,8^ To resolve these challenges, a combined approach for material validation and synthesis optimization is required, wherein comprehensive analysis suites are defined early on and used to confirm the quality of materials produced from new candidate synthesis methods.^9^

Fortunately, nanomaterials are not the first entities to encounter such barriers—pharmaceutical formulations, e.g. tablets consist of complex mixtures of active pharmaceutical ingredients and various excipients,^10^ require stringent control of the composition and purity of each component. To address these challenges, the quality-by-design (QbD) paradigm was developed,^11,12^ involving the definition of critical quality attributes (CQAs)—essential criteria of product performance and attributes—as well as the critical process parameters (CPPs) required to prevent deviation from this specification. Early CQA definition provides a design envelope that can, in turn, be used during reaction engineering and process intensification, enabling a smarter route and process selection while retaining intrinsic compliance with performance and safety requirements.^13^ Given the complex design specifications for nanomaterials,^14^ especially in the emerging field of nanotherapeutics, adoption of QbD stratagems will likely be essential to achieve commercially viable manufacturing routes. The QbD approach has already been applied to perovskite nanomaterials, demonstrating its applicability for optimizing synthesis parameters and ensuring crystalline phase purity.^15^ However, no studies have demonstrated the strategy’s applicability toward noncrystalline (i.e., X-ray inactive) nanomaterials. In this study, we therefore apply the QbD paradigm during noncrystalline nanomaterial process intensification, demonstrating both its applicability to this class of materials and its importance in scale-up to manufacturing.

To test if a QbD approach to noncrystalline nanomaterial synthesis can simplify their process intensification, we apply these techniques to sol–gel silicas, specifically bioinspired silica (BIS) nanomaterials.^16^ Like current industrial precipitated silica manufacturing processes, bioinspired silica materials use fully aqueous solvents, neutral reaction pH (achieved through the use of mineral acids like HCl),^17^ and inorganic sodium silicate precursors, enabling direct incorporation of industrial feedstocks with few (expected) changes of the reaction chemistry involved. These similarities, especially in the synthesis protocol, enable the use of industrial precipitated silica production as a model for a hypothetical bioinspired silica manufacturing process. While this work complements our previous efforts on quantifying and modeling bioinspired silica discovery and design,^18^ their technoeconomic feasibility,^19^ process chemistry,^20^ environmental sustainability,^1,21^ and scale-up,^22^ to our knowledge, no studies exist describing the process intensification and optimization of BIS synthesis toward manufacture.

One notable difference between industrial precipitated silica and bioinspired silica is that the latter uses organic amine additives that facilitate faster reactions as well as aid self-assembly. This enables the synthesis of high-value bioinspired silicas with a large specific surface area, tunable pore sizes, surface chemistry, and the ability to incorporate catalysts or drugs in situ. These additives serve a dual purpose in the synthesis—acting first as proton transfer catalysts during the initial polymerization of silicic acid^23^ and then as a coagulant bridging the electric double layer between colloidal nanoparticles, thus promoting precipitation.^24^ These two roles can be fulfilled by a wide range of amine molecules with consequent ramifications to the BIS microstructure, which has been extensively studied elsewhere.^25^ The use of additives enables much faster and milder reaction conditions for bioinspired silica synthesis cf. current industrial silica production;^19^ however, their incorporation into the silica synthesis can include environmental concerns. For example, the quintessential additive compound pentaethylenehexamine (PEHA) is corrosive and toxic to aquatic life,^26^ meaning that the economic benefits of using additives during bioinspired silica production must be juxtaposed against the risk of accidental environmental release. According to the 12 principles of green chemistry,^27^ these hazards can be minimized in two ways: by the substitution of harmful additives like PEHA with environmentally friendly alternatives during reaction engineering and by reducing the concentration of additives used from stoichiometric levels to catalytic during process intensification. Among a range of amine additives explored to date,^28^ PEHA has shown to offer the best control of silica synthesis and products (very fast reaction kinetics, control over CQAs, ambient reaction temperature, neutral reaction pH). Natural alternatives such as amino acids and bioderived molecules do not provide such control or increased rates, while proteins and polypeptides may offer some control, but they require extremely hazardous chemicals and unsustainable conditions for their synthesis as well as unsustainable conditions/reagents for silica synthesis.^29−31^ Further, when compared to other high-value silica syntheses (e.g., MCM-41, SBA-15, HMS and COK-12 type mesoporous silicas) on the basis of 12 Principles of Green Chemistry, bioinspired silica produced using PEHA has shown excellent overall sustainability.^21^ This reduced environmental impact is attributed to the fast and low-temperature synthesis in water that is enabled by the use of PEHA. Recently, a technology was developed to remove PEHA postsynthesis and its reuse, which further overcomes hazards to the environment.^20,32^ Therefore, considering the complex and multicriteria balance between synthesis conditions, material properties and performance, production costs, and cost-effectiveness,^33^ the use of PEHA provides a clear way forward.^6^ However, in order to optimize the use of PEHA and minimize any environmental risks, a key goal of this study is the reduction of additive concentration in the reaction mixture to the minimum viable concentration.

Aside from the additive compound used, there are several key differences between current bioinspired silica synthesis methods^17^ and current industrial precipitated silica production methods^19^ both in terms of specific reagent sources, how reagents are introduced to the reactor, and the concentration of each component used. In terms of reagent materials, laboratory synthesis techniques traditionally either use alkoxysilane reagents (e.g., tetraethylorthosilicate) or crystalline sodium silicate (Na_2_O·SiO_2_) as a Si source; by contrast, current commercial processes use saturated sodium silicate solutions with a high SiO_2_/Na_2_O molar ratio.^19,34^ Similarly, while laboratory methods may use hydrochloric acid or various organic compounds to adjust the pH, commercial methods generally use sulfuric acid due to greater availability and fewer corrosivity issues than other mineral acids.^34,35^ In terms of reactor design, the order of reagent addition is often overlooked during laboratory-scale synthesis, despite the significant ramifications these choices may have on production in larger batches or on a continuous basis (especially once reagent recycling is considered). Finally, academic research generally uses dilute concentrations and larger relative catalyst concentrations to ensure the ideal behavior of the chemicals in solution, whereas manufacturers will attempt to maximize reagent concentration and minimize catalyst ratios to improve process profitability.

While these challenges appear scientifically trivial, it has been demonstrated for metallic colloids that nanoparticle CQAs are sensitive to these seemingly unimportant methodology variations.^36,37^ This is presumably a consequence of the changed electrolyte environment within the reactor—known to be a highly important and dynamic parameter during batch preparation of sol–gel materials.^38^ By determining sensitivity toward these CPPs at the early stages of process intensification, it will be possible to account for their impact on the CQAs for bioinspired silica, thereby avoiding expensive and time-consuming validation experiments each time process variations are made at a larger scale.

**Figure 1:**
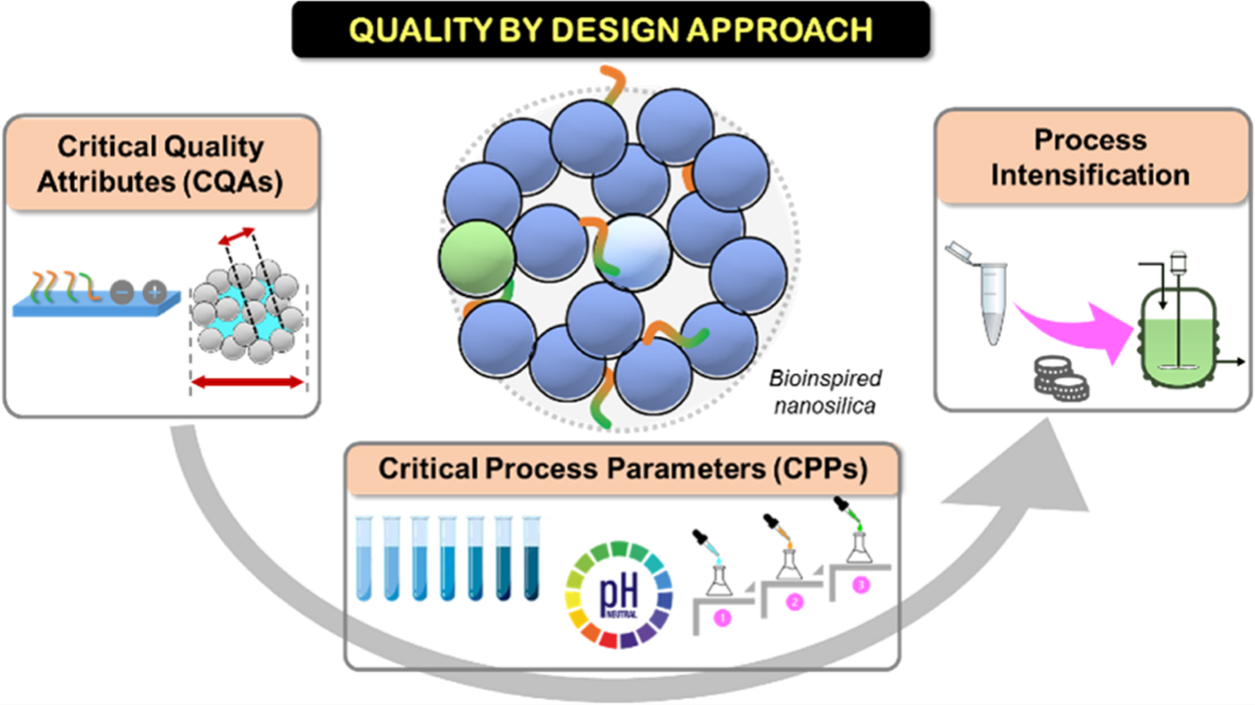
Schematic representation of the three phases of research carried out in this study.

To achieve these goals, we will first define a series of CQAs for bioinspired silica materials, benchmarking material performance, and expected batch-to-batch uncertainty. This analysis will also identify high-sensitivity, low-variability analytical techniques that can best track changes in material quality upon process variations. Once this baseline behavior and analytical techniques have been defined, CPPs will be identified by systematically varying the parameters listed above (i.e., Si source, acid source, order of addition), determining their impact, and hence informing any restrictions on BIS process design. Finally, bioinspired silica synthesis will be intensified by increasing the absolute Si concentration and reducing the relative additive concentration to the greatest extent possible without compromising the identified CQAs. This workflow is shown schematically in Figure 1.

## Experimental Details

### Materials

Sodium silicate pentahydrate (Fisher scientific, technical grade), anhydrous sodium silicate (Fisher scientific, technical grade), and a water glass solution (Crystal 79, kindly supplied by PQ corporation, 3.2:1 SiO_2_/Na_2_O, >27 wt % SiO_2_) were used as received. Pentaethylenehexamine (PEHA, Sigma-Aldrich, technical grade) and a 1 M HCl solution (Fisher Scientific, NIST standard) were used as received. HCl (37.0 wt %) (Honeywell Fluka) and H_2_SO_4_ (Honeywell Fluka, 95–97%) were diluted with deionized water (Millipore, >16 MΩ·m) as required.

For the spectrophotometric analysis, ammonium molybdate tetrahydrate (Fisher Scientific, 98%), oxalic acid (Acros Organics, 98%), anhydrous sodium sulfite (Fisher Scientific, >97%), and *N*-methyl-*p*-aminophenol hemisulfate (*p*-metol, Fisher Scientific, >99%) were used as received.

### Bioinspired Silica Synthesis

“Baseline” bioinspired silica synthesis was performed as described elsewhere.^17,20^ Briefly, sodium silicate (3 mmol) was dissolved in 40 mL of deionized water. Separately, PEHA (0.5 mmol) was dissolved in a further 40 mL of deionized water. These two solutions were mixed in a 180 mL polycarbonate tub, and a further 13 mL of deionized water was added under continuous stirring. To initiate the reaction, ca. 7 mL of a 1 M HCL solution was quickly added. The solution pH was measured continuously, and after 300 s the pH value was 7.00 ± 0.05. Once this time had elapsed, the solid bioinspired silica coagulum was isolated by centrifugation at 5000*g* for 15 min. The solid pellet was redispersed in 50 mL of deionized water and reisolated by centrifugation, repeated twice. Finally, the solid material was dried in an oven overnight at 40 °C. Modifications to this procedure are described in Table 1, and full details of each synthesis procedure (and each execution thereof) are provided in the Supporting Information.

**Table 1:** Table of Target Synthesis Parameters for Each Synthesis Method Used in This Study^a^

method	[Si]/mM	Si/N/-	Si source	H^+^ source	initiator	target pH/-
**A**	30	1	NaSi	HCl	acid	7.00
**B**	30	1	NaSi	HCl	acid	6.75
**C**	30	1	NaSi	HCl	acid	7.15
**D**	30	1	NaSi	H_2_SO_4_	acid	7.00
**E**	30	1	WG	HCl	acid	7.00
**F**	30	1	WG	H_2_SO_4_	acid	7.00
**G**	30	1	WG	H_2_SO_4_	amine	7.00
**H**	30	1	WG	H_2_SO_4_	silicate	7.00
**I**	30	2	NaSi	HCl	acid	7.00
**J**	30	4	NaSi	HCl	acid	7.00
**K**	30	8	NaSi	HCl	acid	7.00
**L**	330	8	WG	H_2_SO_4_	silicate	7.00
**M**	330	16	WG	H_2_SO_4_	silicate	7.00
**N**	660	8	WG	H_2_SO_4_	silicate	7.00
**O**	660	16	WG	H_2_SO_4_	silicate	7.00

a Si source abbreviations are NaSi: sodium silicate pentahydrate and WG: water glass solution.

### Material Analysis

pH measurement was performed either manually using a Hanna HI-1616D electrode or automatically using a Metrohm titrando 902 unit with an Aquatrode pH electrode. Manual recording was performed every 30 s, and automatic recording was performed every 2 s. pH probes were calibrated daily using pH 10.00 and 7.00 buffer solutions (Fisher Scientific, NIST standard solution).

Silica speciation and the extent of the reaction were determined using the silicomolybdate blue spectrophotometric method.^17,39^ A molybdic acid solution was prepared by mixing ammonium molybdate tetrahydrate (51 mmol), a 37 wt % HCL solution (60 mL), and 500 mL of water before diluting with further deionized water to produce a 1 L volumetric solution. A reducing agent solution was produced by mixing oxalic acid (111 mmol), anhydrous sodium sulfite (2 g), *N*-methyl-*p*-aminophenol hemisulfate (10 mmol), and 250 mL of deionized water before further dilution to a 500 mL volumetric solution.

For the analysis, 300 μL of the molybdic acid solution was mixed with 3 mL of deionized water, then with 10 μL of a test solution, whereupon the solution began to turn yellow. After exactly 15 min, 1.6 mL of the reducing agent solution was added and a blue color began to develop. The solution was stored at room temperature for 2–24 h, after which the absorbance at 810 nm was recorded using either a Genesys 10s or Agilent Cary 4000 spectrophotometer.

Nitrogen adsorption isotherms at 77 K were gathered on the materials by using a Micromeritics 3flex manometer. Approximately 100 mg of each sample was weighed into a sample tube, which was then outgassed at 120 °C in a vacuum heating manifold. After at least 3 h of evacuation, samples were reweighed and placed in the 3flex for analysis. Sorption isotherms were gathered according to the Micromeritics “MOFScan” pressure sequence for characterizing microporous–mesoporous solids, modified such that a new isotherm point was gathered after every 30 cm^3^ (STP) of nitrogen was dosed into the analysis tube.

Transmission electron microscopy was carried out in a Philips EM420 TEM microscope equipped with a W filament operating at 120 kV. Images were used to qualitatively explore the materials and aid in the interpretation of scattering analysis.

Ultrasmall-angle X-ray scattering (USAXS) studies were carried out at the Argonne National Laboratory on the beamline 9 ID-C at the Advanced Photon Source.^40,41^ Data reduction and analysis were performed using the IGOR Pro 9 software package using the Irena^42^ and Nika^43^ packages. All samples were fit with a two-level unified fit model to capture primary and secondary silica aggregates,^44^ providing a good fit for all samples (Figure S2). Summary data are provided in Table S2.

## Results and Discussion

### Defining CQAs and Typical Material Behavior

The first priority for intensifying the bioinspired silica synthesis lies in defining “baseline” material properties to compare as a basis for future comparisons. Given the nonspecific nature of this study, we focused on two general CQAs to ascertain material quality: reaction yield and particle textural properties (porosity and particle size).^6^ The yield CQA was determined both from the mass of isolated particles (corrected for an expected raw organic content of ca. 15 wt %)^20^ and from silicomolybdate spectrophotometry measuring residual monomeric and oligomeric silicon species in the reaction mixture.^39,45^ In combination, a full Si mass balance was performed across the reaction, providing insights into the cause for any changes in yield as they arose.^17^ Textural properties of the final powder were measured by nitrogen manometry, transmission electron microscopy, and USAXS. In combination, these techniques provide estimates of the specific surface area, particle and pore size, and aggregation information across multiple length scales including the material mass fractal dimension.^40−43^

**Figure 2:**
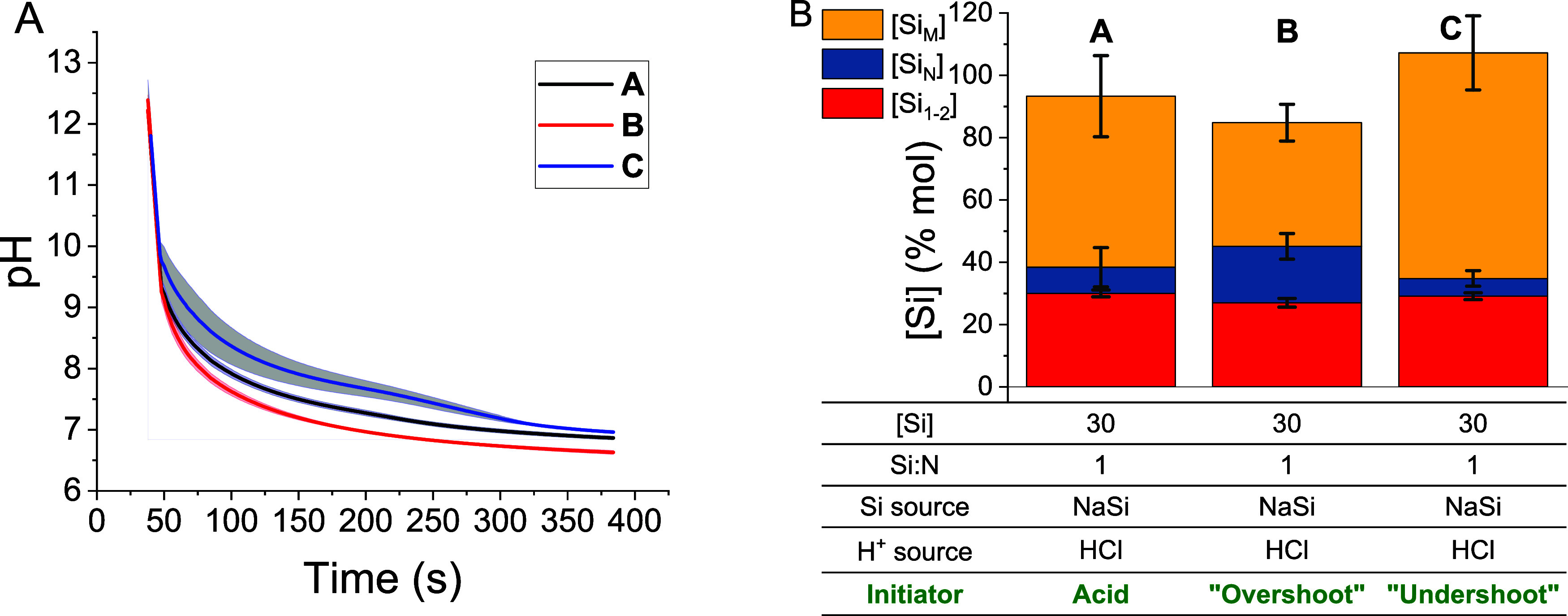
(A) Average (lines) and standard deviation (shaded areas) values for pH progression of syntheses **A**, **B**, and **C** versus time. (B) Si species balances of syntheses **A**, **B**, and **C**. *n* ≥ 3 for all points. Si_N_ and Si_M_ represent the oligomeric and polymeric (precipitated) silicon species, respectively. The parameters changed are shown in green text.

In order to improve on previous estimates of these two CQAs, we expanded the methodology established in reference (17), performing multiple syntheses with an autotitrator to ensure high batch-to-batch consistency. The amount of acid added to the synthesis was then varied by ±2% to mimic unavoidable process variations and thereby perform a sensitivity analysis of the measured properties against the final pH. The three resulting synthesis methods were labeled **A**, **B**, and **C**, representing the “baseline” (final pH = 7.00 ± 0.05), “overshoot” (final pH = 6.73 ± 0.03), and “undershoot” cases, respectively. For the “undershoot” reaction, further acid was added after ca. 180 s to reduce the final pH to 7.18 ± 0.05. Reaction pH as a function of time is shown in Figure 2a.

For the yield CQA, reactions **A**, **B**, and **C** were performed multiple times (*n* ≥ 3), and Si balances were performed around each (Figure 2b). In all cases, the consumption of monomeric or dimeric Si species was approximately equal at ca. 70 ± 1% mol regardless of the amount of acid added (Figure 2b, red). Coagulation of colloidal silica oligomers (Figure 2b, blue) into precipitated solid silica (Figure 2b, yellow) was significantly altered by the change in reaction conditions, however. When additional HCl was added in synthesis **B**, higher quantities of nonprecipitating oligomers were formed (18 ± 4% mol cf. 8 ± 6% mol for **A**). Similarly, when less acid was added in synthesis **C**, fewer oligomers were formed (5 ± 2% mol). Silica precipitate yield changed to compensate for these different oligomer concentrations (Figure 2b, yellow): decreasing from 55 ± 13% mol in synthesis **A** to 40 ± 6% mol in synthesis **B**, and increasing to 71 ± 13% mol in synthesis **C**. We hypothesize that these differences in final Si speciation are caused by changes to the formation pathways as a function of time.^46^ Specifically, when the pH level remains higher for longer (as in synthesis **C**), individual particles will grow larger and scavenge more of the suspended oligomers before coagulating and being removed from the suspension. Conversely, if pH is reduced more quickly, as in synthesis **B**, the particles will have less opportunity to grow before coagulation is induced and therefore react with fewer colloidal oligomers. Regardless, these data provide useful bounds for the yield CQA based on synthesis **A** and highlight the sensitivity of BIS synthesis to small changes in the reaction pH profile. Further, the results also demonstrate the efficacy of silicomolybdate spectrophotometry in tracking changes to the progress of the reaction.

**Figure 3:**
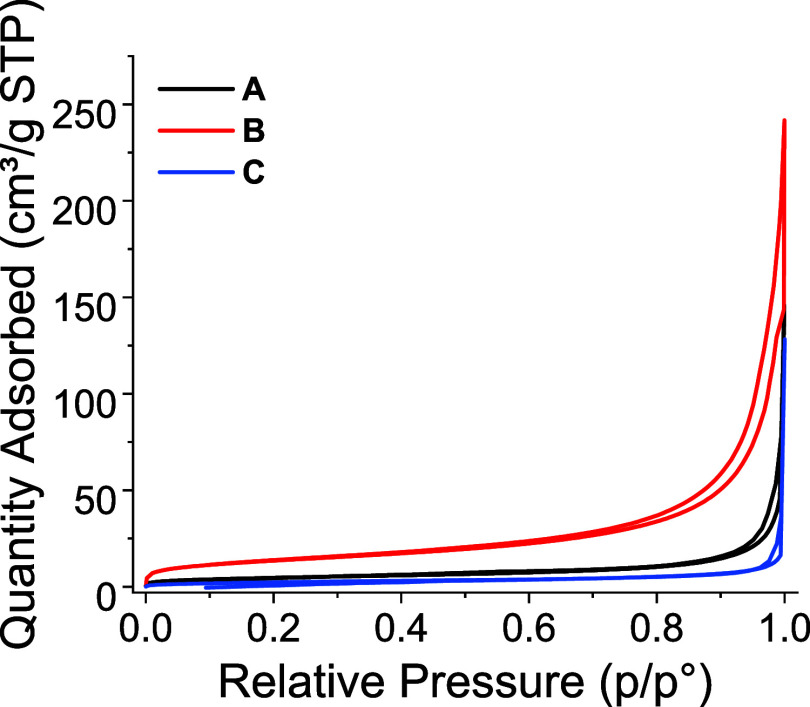
Nitrogen adsorption isotherms for materials **A**, **B**, and **C**.

In the textural properties of CQA, multiple analytical methods were identified as candidates for assessing particle quality: USAXS, transmission electron microscopy (TEM), and nitrogen manometry. To choose the most appropriate technique for routine analysis, we measured textural properties for materials **A**, **B**, and **C** in turn. From the nitrogen manometry (Figure 3), specific surface areas of 16, 47, and 9 m^2^/g were recorded for **A**, **B**, and **C**, respectively, consistent with previous findings for nonpurified bioinspired silica materials.^18^ Similarly, small differences were measured in the pore-size distributions and total pore volume (Figure S2 and Table S1). These results provide a precise specification of “baseline” behavior compared to bioinspired silica materials made with other silica sources,^47^ polymeric amine additives,^48^ or where the additives have been removed.^20^ Therefore, deviation from these low surface area values indicates a large change in the progress of the reaction rather than uncontrollable batch-to-batch variation.

**Figure 4:**
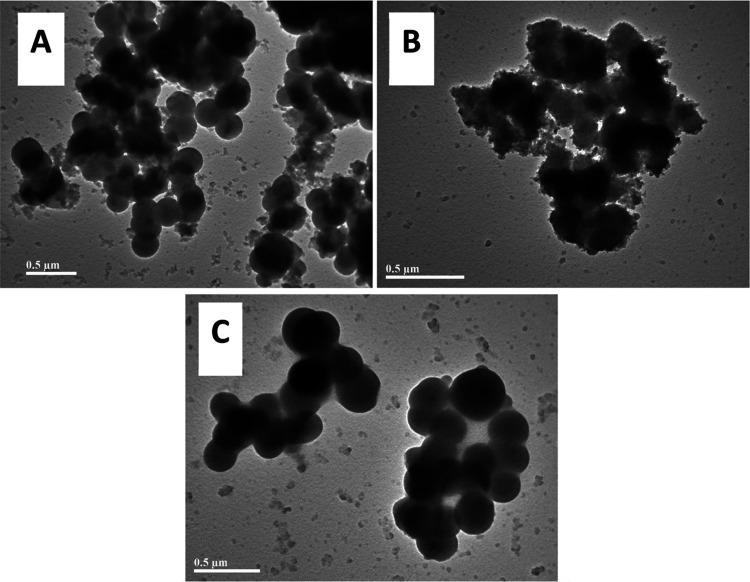
Representative TEM micrographs of materials **A**, **B**, and **C**.

Unlike porosity, changes in titer volume between syntheses **A**, **B**, and **C** did not reliably cause changes to the particle structure or morphology. Particles were analyzed with both USAXS (Table S2, and Figures S2–S4) and TEM (Figure 4); however, no statistically reliable differences in the structure or morphology could be identified through either method. While certain USAXS curves aligned reasonably well with the two-level unified fit model, many displayed parameters that are physically improbable. For instance, some fitted Porod’s law prefactors exceeded that of any conceivable polydisperse or asymmetric particle with a singular radius of gyration. Such anomalies can be traced back to the particle geometry, specifically, fractals composed of correlated polydisperse spheres. Support for this assertion can be drawn from the TEM images (Figure 4) and further deduced from the USAXS spectra. Notably, the spectra reveal characteristic replicated “humps” indicative of sphere scattering.

In the few instances where the USAXS fitting provided physically sensible values, all syntheses showed similar primary particle sizes ranging from ca. 180 to 200 nm, with consistently high polydispersity seen across all samples (with a log-normal variance of ca. 0.3 for all materials studied). Surface properties, such as the roughness of the particles, did not appear to fluctuate significantly, as the fractal dimension remained between ca. 2.2 and 2.8 for all three syntheses. As a result, it was decided that neither USAXS nor TEM would be suitable for the purposes of this study as the particles produced were too polydisperse to statistically differentiate with currently available scattering models.

From the trial experiments conducted, we were able to determine baseline behavior for both silica yield and textural properties. We found that silicomolybdate colorimetry and nitrogen manometry were both sensitive enough to identify small, deliberate variations to the synthesis method and specific enough to demonstrate the similarities between them. This allowed us to generate reasonable confidence intervals for both CQAs before making larger changes to the synthesis methods. Should intensified synthesis routes produce materials with similar properties, this would indicate their application-specific performance will match that of the “baseline” materials.

### Determining CPPs for Batch Bioinspired Silica Synthesis

In terms of chemical reagents, three CPPs have been identified by comparing current bioinspired syntheses against industrial precipitated silica production methods: Si source, acid source, and order of reagent addition. First, we systematically substituted the reagents used in the synthesis—from HCl to H_2_SO_4_ and from sodium silicate pentahydrate to sodium-reduced water glass. As both substitutions affected the background electrolyte concentration in the reaction mixture, there was a strong possibility of confounding effects on the synthesis. Therefore, we used a 2^2^ factorial design to capture both the direct effects and interactions of reagent substitution on the BIS synthesis.

**Figure 5:**
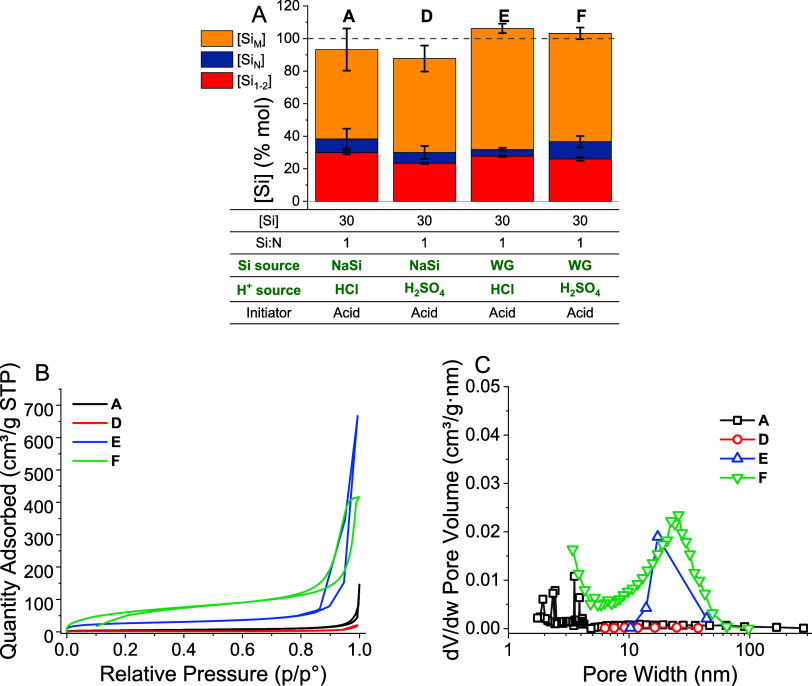
(A) Effect of changing Si and acid sources on the silica yield. The parameters changed are shown in green text. (B) Nitrogen adsorption isotherms and (C) BJH pore-size distributions of materials **A**, **D**, **E**, and **F**.

As a result, we developed syntheses **D** (only switching HCl with H_2_SO_4_), **E** (only switching sodium silicate pentahydrate with water glass), and **F** (switching both the acid and silicate), which showed only slight changes to the silica yield CQA (Figure 5a, compared against baseline synthesis **A**). By performing ANOVA on the reactions, only the choice of the Si source had a significant effect on the final solid yield (*p* = 0.044), increasing it by ca. 10% mol. Changing the acid source to H_2_SO_4_ slightly increased monomer consumption by ca. 5% mol (*p* = 2 × 10^–4^), while changing both acid and Si sources approximately reversed this effect (*p* = 0.009). The increased solid yield in synthesis **E** is similar to that seen with the higher-pH synthesis **C**, and presumably occurs for the same reason—reduced [Na^+^] in solution delays coagulation, enabling more efficient oligomer scavenging. Conversely, the increased monomer consumption seen in synthesis **D** may be a result of the softer sulfate anion increasing the rate of proton transfer catalysis during initial Si polymerization.^49^ Overall, despite the statistical significance, changes to the reaction progress upon introduction of industrial reagents were minimal, indicating that overall silica quality was unchanged.

Despite the relatively low impact of the reagent choice on the yield CQA, textural properties changed significantly upon substitution of the silica source (Figure 5B,C). While substitution of the mineral acid in synthesis **D** had no effect on the BIS-specific surface area (decreasing from 16 m^2^/g in material **A** to 8 m^2^/g), changing the Si source to water glass created much more porous silicas (81 m^2^/g and 240 ± 16 m^2^/g for syntheses **E** and **F**, respectively). Furthermore, broad mesopores between ca. 10 and 20 nm in width appeared when using water glass, which we ascribe to interstitial voids within the silica aggregate, the diameter of which is known to correlate with primary particle size.^50^ We again assume this is due to the nature of coagulation during the reaction due to reduced [Na^+^] in the reaction mixture. Therefore, although changes to the overall yield were minimal, the material quality was significantly altered by changing the Si source. Further dedicated studies would therefore be required to determine the effect of reagent substitution on application-specific material properties, e.g. drug release.

**Figure 6:**
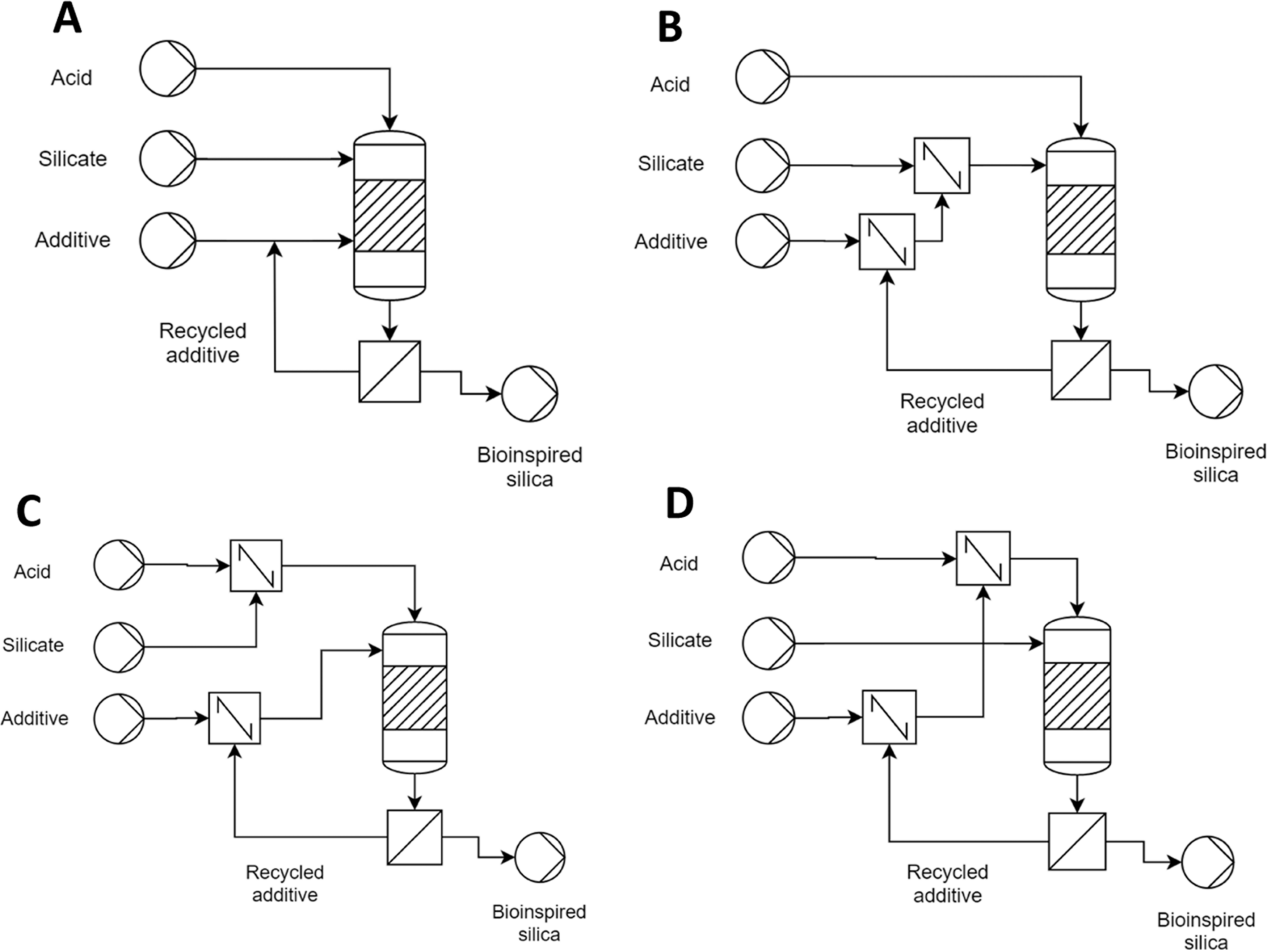
Simplified process flow diagrams for silica synthesis, demonstrating the potential order-of-addition combinations in a scaled-up synthesis: (A) simultaneous addition of all reagents, (B) acid initiation by premixing of the Si source and the additive, (C) additive initiation by premixing of the Si source and acid, and (D) silicate initiation by premixing of acid and the additive.

In addition to the change of reagents, a second CPP identified was the reagent order of addition. As the reaction is only initiated when the third reagent is introduced, there are several combinations of reagent addition that may be realistically employed (Figure 6). Simultaneous, independent addition of all three reagents (Figure 6a) is impractical during lab-scale preparation; therefore, previously the reaction was initiated by addition of the acid component (corresponding to Figure 6b). Accordingly, to assess the importance of the reagent order of addition, acid-initiated synthesis **F** was modified to give amine-initiated synthesis **G** (corresponding to Figure 6c) and silicate-initiated synthesis **H** (Figure 6d).

**Figure 7:**
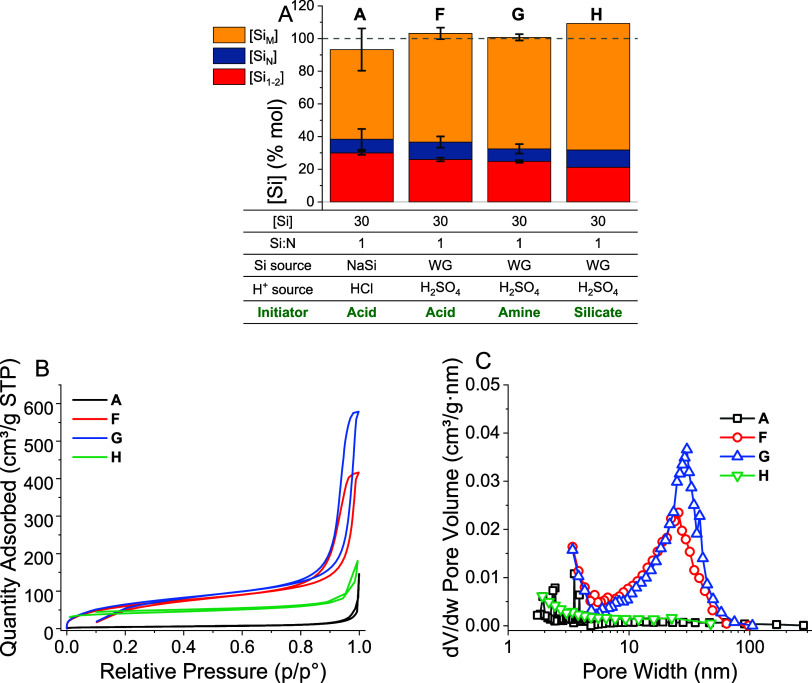
(A) Effect of changing order of addition on silica yield CQA. *n* = 3 for syntheses **A**, **F**, and **G**, and 1 for synthesis **H**. The parameters changed are shown in green text. (B) Representative nitrogen adsorption isotherms and (C) BJH pore-size distributions for materials **A**, **F**, **G**, and **H**.

The relative reaction yields for syntheses **F**, **G**, and **H** are shown in Figure 7a. Again, only slight changes to the yield could be identified from ANOVA analysis: monomer consumption increased by ca. 1.5% mol when the reaction was initiated by silicate cf. acid initiation (*p* = 0.01), and solids yield correspondingly increased by ca. 3% mol (*p* = 0.03). However, as no large changes to the reaction yield were identified, we concluded that changing the order of addition did not affect the silica yield.

As all of materials **F**, **G**, and **H** were synthesized using water glass rather than sodium silicate, all had much larger specific surface areas than material **A**. Regardless, the silica textural properties did appear dependent on the reaction’s order of addition (Figure 7b). While material **G** had a similar surface area to material **F** (230 ± 17 and 240 ± 16 m^2^/g, respectively), material **H** had an intermediate surface area of 148 m^2^/g. In terms of pore-size distribution, both materials **F** and **G** had significant mesopore volume with an average pore diameter of ca. 30 nm (Figure 7c); however, material **H** showed no such mesoporosity, again supporting the notion that the order of addition could have a significant effect on bioinspired silica coagulation.

The effect of the amine protonation state on the eventual silica structure has previously been reported both in bioinspired^23,24^ and other amine-templated silica systems;^51^ however, to our knowledge, this is the first case where mesoporous bioinspired silica can be produced without any changes to the synthesis chemistry and simply by controlling the processing. While further investigation is needed to explain the change in textural behavior with different orders of reagent addition, the continued high yields of the reaction indicate that the synthesis is robust to the process changes required for scale-up.

### Process Intensification

The next challenge lies in intensifying the synthesis to match the current commercial processes. Industrially, precipitated silicas are made at concentrations between 1 and 10% wt in solution, cf. 0.18% wt used during syntheses **A–H**. Further, the addition of the amine additive PEHA in stoichiometric quantities significantly increases the environmental impact and direct processing costs of bioinspired silica synthesis;^19^ therefore, its concentration should be minimized. These parameters have been recently explored in limited terms by Dewulf et al. using factorial design,^18^ demonstrating the robustness of bioinspired silica synthesis to changes in reagent concentrations in general. Here, we extend this approach to explore the limits of silica and additive concentration to minimize the specific cost and environmental impact of bioinspired silica without compromising the two identified CQAs.

**Figure 8:**
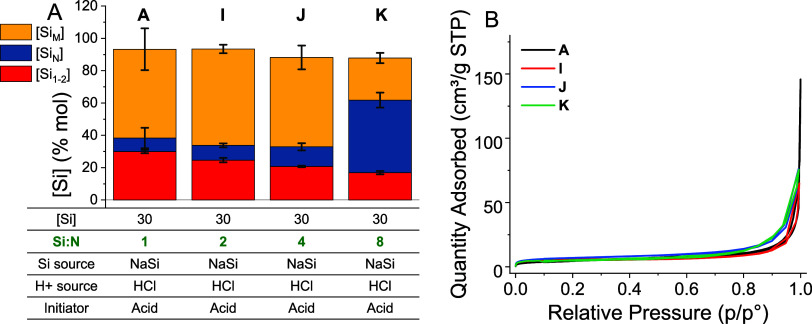
(A) Effect of the Si/N ratio on the bioinspired silica yield. The parameters changed are shown in green text. (B) Nitrogen adsorption isotherms of materials **A**, **I**, **J**, and **K**.

Our first step in process intensification was to reduce the additive concentration relative to the silica concentration (i.e. the Si/N molar ratio). To this end, we developed syntheses **I**, **J**, and **K** with Si/N ratios of 2, 4, and 8, respectively (Figure 8a). Previously, increasing Si/N from 0.5 to 2 was shown to have a small negative effect on the precipitate yield,^18^ and separately that increasing Si/N beyond 4 drastically increased bioinspired silica gelation time owing to reduced ability of additives to flocculate silica colloids.^24^ In terms of yields, our results largely agree with these previous studies: solid silica yield was not significantly affected up to Si/N = 4, but increasing Si/N to 8 (synthesis **K**) dramatically reduced the solid yield from 58 ± 13 to 33 ± 13% mol, with a corresponding increase in oligomeric silica colloid formation.

Looking beyond the solid yield, monomer consumption increased approximately linearly with Si/N. This may be a result of increased stabilization of colloidal oligomers at low additive concentrations; coagulation of silica colloids reduces their activity in solution, thus lowering the rate of monomer consumption for particle growth. Regardless, these findings demonstrate that bioinspired silica can be synthesized at very high Si/N ratios (up to 4), significantly reducing the associated cost of using amine additives. In terms of the material’s textural quality, the Si/N ratio had no appreciable effect—surface areas for materials **A**, **I**, **J**, and **K** were all between 15 and 25 m^2^/g, and neither microporosity nor mesoporosity was measured in the pore-size distributions.

In addition to reducing the concentration of catalytic additives within the reaction, specific costs of synthesis can be minimized by increasing the initial [Si]. This is particularly relevant in the case of bioinspired silica, as the cost of water treatment has been demonstrated to be significantly higher than the equivalent cost for current industrial precipitated silicas.^19^ Previous studies have synthesized bioinspired silica at concentrations up to 90 mM [Si],^52,53^ far lower than current manufacturing concentrations of 1–10% wt (ca. 165–1650 mM [Si]). To assess the limit of [Si] concentration during bioinspired silica synthesis, we sequentially increased the concentration from 30 mM to 330 and 660 mM (i.e., 2 and 4% wt). One immediate consequence was a higher viscosity, requiring the use of an overhead stirrer rather than a magnetic stir bar to adequately mix the reaction mixture. At the highest concentration, the reaction vessel was also manually agitated to prevent the formation of a solid crust on the surface. Further, due to the practical ramifications of the high concentration, amine initiation of the reaction was no longer possible at 660 mM as silica gel formed prematurely during the mixing of the silicate solution with acid.

**Figure 9:**
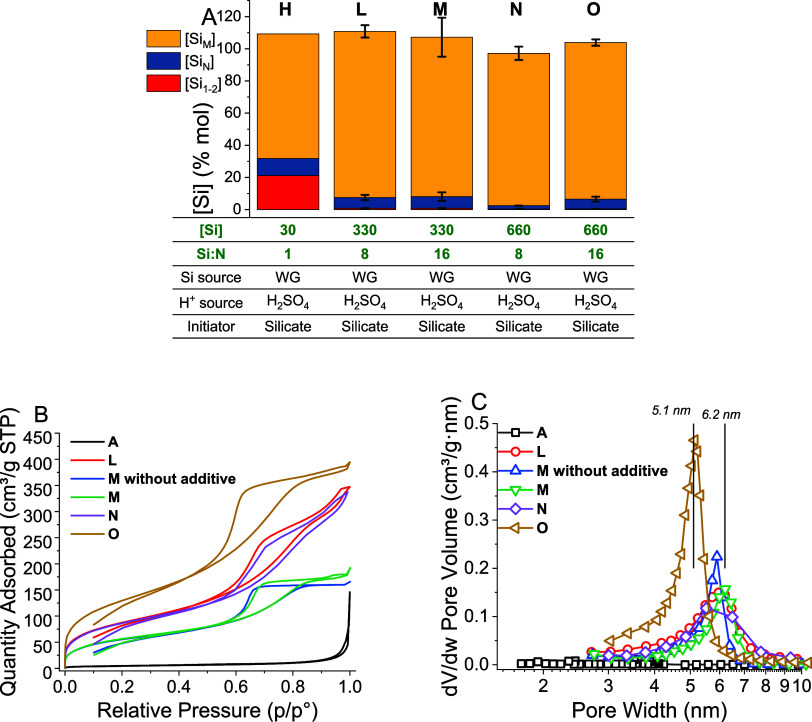
(A) Effect of [Si] and the Si/N ratio at high concentrations. The parameters changed are shown in green text. (B) Representative nitrogen adsorption isotherms of high-concentration bioinspired silica materials **L**, **M**, **N**, and **O** compared against baseline material **A** and material **M** synthesized with no bioinspired additive. (C) Corresponding BJH pore-size distributions of high-concentration BIS materials.

As a result, we focused on higher concentration analogues of silicate-initiated synthesis **H** (Figure 9a), using a further 2^2^ factorial design across the Si/N and [Si] variables, creating syntheses **L**, **M**, **N**, and **O**. In all cases, higher [Si] significantly increased the relative precipitate yield compared to the original concentration, from ca. 66 to ca. 98% mol. Monomer consumption consequently increased from 70 ± 1% mol for material **A** to >99% mol for materials **L**, **M**, **N**, and **O**, corresponding to an absolute unreacted monomer concentration of 2.8 ± 0.2 mM for all four high-concentration syntheses. This constant concentration indicates that silica polymerization reached thermodynamic completion in these syntheses. When comparing the high-concentration synthesis methods using ANOVA, none of the reaction parameters had any statistically significant effect on the precipitate yield. Monomer consumption increased with [Si] (*p* < 0.001) but was unaffected either by the Si/N ratio or the interaction between [Si] and Si/N.

Final oligomer concentration remained similar to synthesis **A** in most cases: 6 ± 1% mol for synthesis **L**, **M**, and **O**, but reduced to 2.0 ± 0.01% mol in the case of synthesis **N**. ANOVA analysis indicated that the final oligomer concentration was decreased by [Si] but increased by both the Si/N ratio and the Si/N–[Si] interaction. Evidently, while the elevated [Si] was enough to drive the silica polymerization reaction to completion, coagulation was still reduced in some cases by the relative scarcity of the amine additive.

Overall, the high-concentration experiments demonstrate that increased [Si] dramatically improves the yield, counteracting any negative effects of increasing Si/N up to 16. By implementing these changes, we were able to increase the specific silica yield from ca. 1.1 g/L to ca. 38 g/L while simultaneously reducing the additive requirement from ca. 1 to ca. 0.04 g(additive)/g(silica). This optimization of bioinspired silica synthesis with regards to the yield overcomes many of the previously identified challenges to scaling up the material, simultaneously reducing water intensity and the cost of using and handling amine additives.^19^

When considering the textural properties CQA, it becomes clear that increasing the [Si] and Si/N ratio has significant effects on the pore structure of the materials. All samples synthesized at elevated concentrations had increased surface areas compared to synthesis **A**: 318 m^2^/g for material **L**; 211 m^2^/g for synthesis **M**; 321 m^2^/g for material **N**; and 375 m^2^/g for material **O**. Furthermore, when material **M** was synthesized with no amine additive, a specific surface area of 208 m^2^/g was recorded. This finding indicates that while the majority of the additional surface area could be attributed to the increased [Si], the inclusion of amine additive contributed to the porosity. As shown in Figure 9b,c, materials **L**, **M**, **N**, and **O** show significant mesoporosity and hysteresis in the region between 0.5 and 0.8 relative pressure, similar to the behavior of material **M** synthesized with no additive. This corresponds to mesopores with a diameter of 5–6 nm, cf. 6 nm for synthesis **M** synthesized with no additive (Figure 9c). Interestingly, when the synthesis was scaled up to 40 L batches at moderate [Si], high yields were maintained, with the porosity being unaffected (data not shown).

When compared with the baseline bioinspired silica **A**, these findings demonstrate a significant change from the initial textural properties. While potentially advantageous for a variety of applications, the mesopores found in materials **L**, **M**, **N**, and **O** demonstrate that increased [Si] affects the silica formation pathway, which, in turn, influences the porosity and presumably other performance-related properties. Further investigation is clearly required for these high-concentration synthesis methods to determine the effect of process intensification on application-specific performance metrics.

While all 4 of the high-concentration silica materials had similar yield and textural properties, increasing the Si/N ratio to 16 (i.e., materials **M** and **O**) led to significant gelation rather than coagulation of the reaction mixture (Figure S5). As a result, the gel could not be reliably separated from the sol by centrifugation due to the reduced density difference compared to a coagulum,^54^ leading to longer dewatering and drying times. Detailed analysis of the effect of gelation vs coagulation on both the process economics and material performance is outside the scope of the current study; however, this finding underlines the challenges present during nanomaterial intensification and the need for systematic investigation at all stages. These findings highlight the need for focusing on process engineering research when scaling up nanomaterial synthesis, with a particular focus on process equipment and downstream processing.

## Conclusions

In this study, we applied the QbD paradigm to the synthesis of bioinspired silica nanomaterials, observing how process decisions, feedstock concentration, and additive concentration affect material quality. Using minor modifications to the previously established synthesis method, we analyzed several material properties to study the variations caused by deliberate changes to the synthesis methodology. We found that the broad distribution of particle size and mass fractal dimension measured by USAXS and TEM did not allow a distinction between different synthesis routes. In contrast, silica speciation and bulk textural properties (i.e., BET surface area and pore-size distribution) were more sensitive to minor changes in the synthesis conditions than interbatch variation. Therefore, we defined yield and bulk porosity as CQAs of the synthesis, where any change in the CQAs with different synthesis procedures would indicate significantly altered materials.

We then defined several critical process parameters to the production of bioinspired silica synthesis at scale—use of industry-friendly reagents and changing the order of reagent addition—and assessed their impact on the CQAs. While the silica yield was unaffected by reagent substitution and changed order of reagent addition, switching to industrial reagents introduced broad mesopores in the resultant materials, which is of interest for high-value applications. We posit that this is a result of lower ionic strengths and softer counterions in the reaction mixture, leading to delayed coagulation and, in turn, larger interstitial pores in the eventual aggregates. Once these modifications had been incorporated, we intensified the synthesis at a small scale by both increasing the Si/N ratio and increasing absolute [Si]. We found that increasing the Si/N ratio above 4 significantly reduced the silica yield by stabilizing colloidal silica in solution. Increasing the initial [Si] in line with current industrial processes broadly counteracted this effect, leading to much higher yields of ca. 98%mol, but with significantly altered textural properties. In terms of textural properties, high [Si] materials had mesopores in the range of 5–6 nm, similar to silica gel formed in the absence of amine additives. As a result, further work is required to investigate the effect of the measured textural changes on the material performance for specific applications. The gelation at very high Si/N ratios rather than coagulation was also observed at high [Si].

Overall, we successfully applied all of the changes to bioinspired synthesis to make it compatible with existing industrial silica manufacturing. We increased the silica-specific yield 35 times and reduced the additive intensity 25 times, resulting in a significant reduction in environmental impact, synthesis cost, and waste production. In combination with previously reported results (e.g., synthesis conditions, properties and performance, costs and benefit), we conclude that bioinspired silica provides a greener alternative to manufacturing high-value silicas, despite the use of amine additives. Nevertheless, the dependence of bioinspired silica CQAs on some processing parameters demonstrates the need for future research on the process intensification of nanomaterial synthesis methods. Specifically, understanding how critical processing parameters like reagent chemistry interact with silica formation pathways and CQAs will help to provide significant insights for process scale-up and commercialization. Such an understanding can also enable process flexibility where materials of different grades can be produced using the same platform, with minor variations to the processing methods.

## Supporting Information Available

Details of all synthesis, yield, and surface area information used in this study and pore-size distribution and fitted USAXS data for selected samples (PDF)
