# Supplementary material for: Quality-by-Design Approach to Process Intensification of Bioinspired Silica Synthesis
Source: ACS Sustain Chem Eng. 2024 Mar 8;12(12):4900–11. doi: 10.1021/acssuschemeng.3c07624 (PMC10966740; doi:10.1021/acssuschemeng.3c07624)
Supplement: Supplementary file 1 — sc3c07624_si_001.pdf [file sc3c07624_si_001.pdf]

A Quality-by-Design approach to process intensification of bioinspired silica synthesis

Joseph R. H. Manning[1,2], Carlos Brambila[1], Kabir Rishi[3], Gregory Beaucage[3], Gemma-Louise Davies[2], and Siddharth V. Patwardhan*[1]
[1] Green Nanomaterials Research Group, Department of Chemical and Biological Engineering, University of Sheffield, Sheffield, S1 3JD, United Kingdom
[2] Department of Chemistry, University College London, London, WC1H 0AJ, United Kingdom

[3]Department of Chemical and Materials Engineering, University of Cincinnati, Cincinnati, Ohio, 45221, United States of America
* corresponding author: s.patwardhan@sheffield.ac.uk

Summary

Page S2:
Table S1 – Complete table of synthesis parameters, yield characteristics, and specific surface area values

Page S3:
Figure S1 – Graphs of BJH pore size distribution for materials **A**, **B**, **C**, **I**, **J**, and **K**
Figure S2 – Raw USAXS fits for materials **A**, **B**, and **C**, measured in triplicate

Page S4:
Table S2 – Table of USAXS unified fit parameters for materials **A**, **B**, and **C**
Figure S3 – Photograph of gel formation during high-concentration synthesis **O**

Table S1 – Summary of all synthesis, yield and surface area information used in this study.

Synthesis method	*[Si] / mM*	*Si:N / -*	*Si source*	*H^+ source*	*Initiator*	*Final pH / -*	*Monomer yield / % mol (mM)*	*Oligomer yield / % mol (mM)*	*Precipitate yield / % mol (mM)*	*SSA^b / m^2/g*	*$d_{pore}{}^b$ / nm*	*$V_{pore}{}^b$ / cm^3/g*
A	30	1	NaSi	HCl	Acid	7.00 ± 0.05	30.0 ± 1.1 (8.99 ± 0.32)	8.4 ± 6.3 (2.51 ± 1.89)	54.9 ± 13.0 (16.48 ± 3.89)	16	-	0.07
B	30	1	NaSi	HCl	Acid	6.73 ± 0.03	27.0 ± 1.4 (8.11 ± 0.43)	18.1 ± 4.1 (5.43 ± 1.22)	39.7 ± 5.9 (11.9 ± 1.77)	47	-	0.22
C	30	1	NaSi	HCl	Acid	7.18 ± 0.05	29.1 ± 1.1 (8.72 ± 0.34)	5.7 ± 2.5 (1.72 ± 0.76)	72.4 ± 11.9 (21.71 ± 3.57)	9	-	0.025
D	30	1	NaSi	H_2SO_4	Acid	7.00 ± 0.05	23.5 ± 0.5 (7.04 ± 0.16)	6.5 ± 4.0 (1.96 ± 1.21)	57.7 ± 8.0 (17.31 ± 2.39)	8	-	0.03
E	30	1	WG	HCl	Acid	7.00 ± 0.05	27.7 ± 0.6 (8.32 ± 0.17)	4.1 ± 1.1 (1.22 ± 0.34)	74.4 ± 2.9 (22.3 ± 0.87)	81	-	1.04
F	30	1	WG	H_2SO_4	Acid	7.00 ± 0.05	26.0 ± 1.1 (7.79 ± 0.32)	10.7 ± 3.4 (3.21 ± 1.03)	66.5 ± 3.6 (19.95 ± 1.07)	240 ± 16[c]	25-30[c]	0.76 ± 0.12
G	30	1	WG	H_2SO_4	Amine	7.00 ± 0.05	24.8 ± 0.08 (7.44 ± 0.23)	7.7 ± 2.9 (2.32 ± 0.86)	68.3 ± 2.0 (20.48 ± 0.60)	230 ± 17[d]	25-30[d]	0.67 ± 0.04
H[a]	30	1	WG	H_2SO_4	Silicate	7.00 ± 0.05	21.1 (6.35)	10.7 (3.20)	77.4 (23.22)	148	-	0.24
I	30	2	NaSi	HCl	Acid	7.00 ± 0.05	24.7 ± 1.4 (7.41 ± 0.41)	9.1 ± 1.2 (2.73 ± 0.37)	59.7 ± 2.7 (17.90 ± 0.79)	21	-	0.06
J	30	4	NaSi	HCl	Acid	7.00 ± 0.05	20.8 ± 4.7 (6.23 ± 0.14)	12.1 ± 2.2 (3.64 ± 0.66)	55.3 ± 7.4 (16.60 ± 2.21)	20	-	0.11
K	30	8	NaSi	HCl	Acid	7.00 ± 0.05	16.9 ± 1.0 (5.08 ± 0.31)	34.7 ± 12.5 (10.42 ± 3.74)	32.9 ± 13.9 (9.86 ± 4.18)	20	-	0.12
L	330	8	WG	H_2SO_4	Silicate	7.00 ± 0.05	0.8 ± 0.0 (2.70 ± 0.16)	6.7 ± 1.6 (22.13 ± 5.32)	103.3 ± 3.8 (340.93 ± 12.65)	318	5.9	0.54
M	330	16	WG	H_2SO_4	Silicate	7.00 ± 0.05	0.9 ± 0.1 (2.92 ± 0.37)	7.2 ± 2.6 (23.75 ± 8.74)	99.1 ± 12.1 (327.02 ± 39.93)	211	6.2	0.28
N	660	8	WG	H_2SO_4	Silicate	7.00 ± 0.05	0.4 ± 0.0 (2.92 ± 0.37)	2.0 ± 0.0 (23.75 ± 8.74)	94.8 ± 4.1 (625.37 ± 27.29)	321	6.0	0.52
O	660	16	WG	H_2SO_4	Silicate	7.00 ± 0.05	0.4 ± 0.0 (2.87 ± 0.10)	6.0 ± 1.5 (40.02 ± 10.22)	97.4 ± 1.9 (642.67 ± 12.86)	475	5.1	0.59

(a) n=1; (b) n=1 unless otherwise noted; (c) n=2; (d) n=3

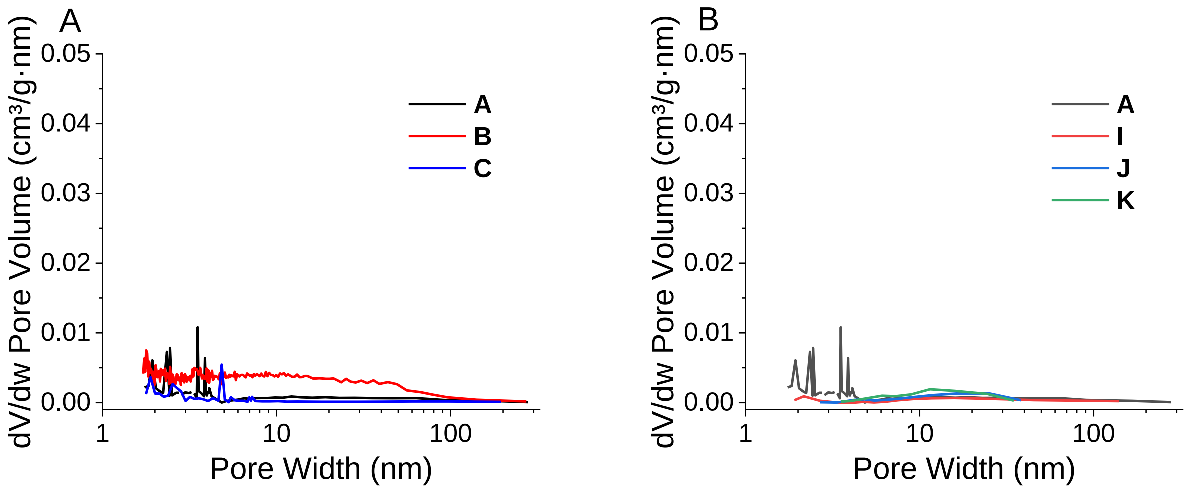

*Figure S1 –BJH pore-size distributions for (a) materials **A**, **B**, and **C**, (b) materials **A**, **I**, **J**, and **K**.*

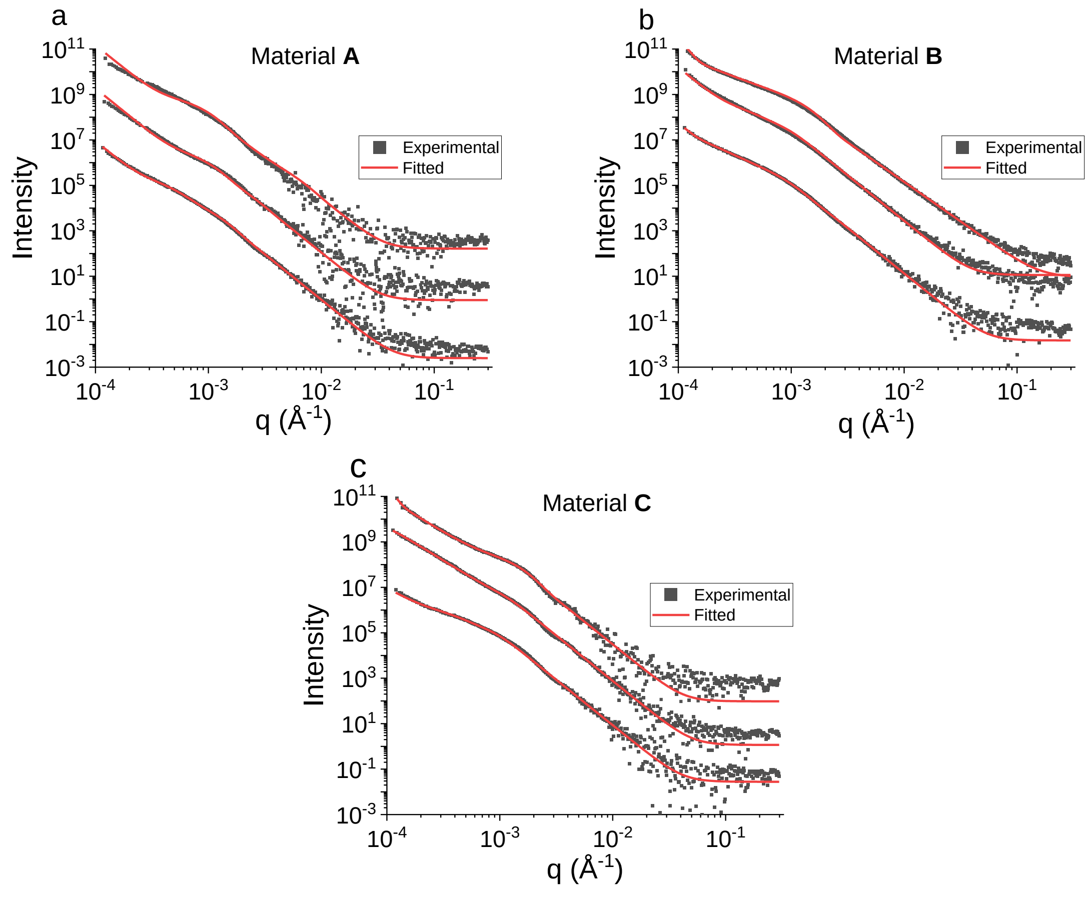

*Figure S2 - Fitted USAXS data (measured in triplicate) for BIS created using synthesis (a) **A**, (b) **B**, and (c) **C**. Repeat measurements are vertically offset to ease visual interpretation.*

Table S2 - averaged fitted USAXS data for synthesis methods A-C using the unified fit method.

Synthesis method	*G / cm⁻¹*	*R_g / Å*	*$\sigma_{lognormal}$ / -*	*B / -*	*P / -*	*S/V / m^2/cm^3*	*d_p / nm*
Level 1							
A	$8.3 \times 10^4 \pm 5.1 \times 10^4$	1800 ± 200	0.33 ± 0.03	$4.9 \times 10^{-8} \pm 4.2 \times 10^{-8}$	-4	26 ± 5	241 ± 51
B	$1.3 \times 10^5 \pm 1.8 \times 10^5$	1400 ± 500	0.36 ± 0.08	$7.7 \times 10^{-7} \pm 9.6 \times 10^{-7}$	-4	64 ± 27	226 ± 15
C	$4.4 \times 10^5 \pm 6.7 \times 10^5$	2100 ± 900	0.33 ± 0.03	$8.9 \times 10^{-8} \pm 6.1 \times 10^{-8}$	-4	26 ± 5	208 ± 22

Synthesis method	*G / cm^{-1}*	*R_g/Å*	*B / -*	*d_f / -*	*z / -*
Level 2					
A	$7.9 \times 10^7 \pm 8.0 \times 10^7$	17,100 ± 5000	$1.0 \times 10^{-3} \pm 1.5 \times 10^{-3}$	2.8 ± 0.2	1400 ± 1300
B	$4.0 \times 10^8 \pm 1.2 \times 10^8$	22,300 ± 3800	$7.4 \times 10^{-1} \pm 1.3 \times 10^0$	2.2 ± 0.3	17,400 ± 19,900
C	$4.0 \times 10^8 \pm 4.4 \times 10^8$	23,100 ± 7,300	$1.1 \times 10^{-1} \pm 1.9 \times 10^{-1}$	2.7 ± 0.6	9900 ± 15100

G is the Guinier prefactor, R_g is the radius of gyration of the primary particles in level 1 and aggregates in level 2, *B* is the power-law prefactor at intermediate *q* which is either surface fractal (>3) or mass fractal (<3). *P* is the slope of the plot at high *q*, which should equal to -4 according to Porod's law. *S/V* is the surface to volume ratio for the primary particles, d_p is the Sauter mean diameter related to *V/S* for these particles, d_f is the mass fractal dimension of the silica aggregates, and *z* is their degree of aggregation. Errors are one standard deviation around the mean for all USAXS fittings shown in Figure S2; n=3 for all samples.

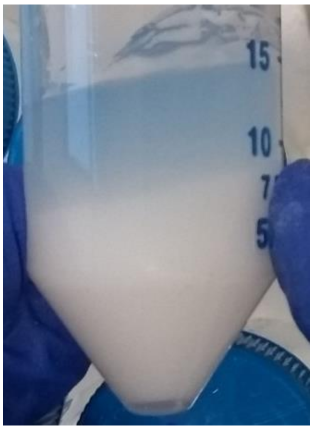

*Figure S3 – photograph of a 50 mL centrifuge tube containing a mixture of precipitated BIS (white, opaque) and gelled BIS (translucent, bluish), produced using synthesis **O**.*
